# Consumption of beef sandwiches in the United States and contributions to intake of energy and select nutrients

**DOI:** 10.3389/fnut.2024.1355490

**Published:** 2024-06-10

**Authors:** Mary M. Murphy, Maribeth M. Anderson, Xiaoyu Bi

**Affiliations:** Center for Chemical Regulation & Food Safety, Exponent, Inc., Washington, DC, United States

**Keywords:** nutrition surveys, National Health and Nutrition Examination Survey, sandwiches, beef, dietary patterns, burgers

## Abstract

**Introduction:**

Sandwiches are commonly consumed in the United States. This study summarizes contributions of beef sandwiches to energy and select nutrient intakes.

**Methods:**

Beef sandwiches were categorized as beef burger sandwiches (hamburgers or cheeseburgers) and non-burger beef sandwiches. *Per capita* and per user consumption of beef sandwiches (total and by type) and contributions to total nutrient intakes from beef and non-beef sandwich components were estimated for the population ages ≥2 years (*n* = 15,984) participating in WWEIA/NHANES, 2013–2016.

**Results:**

On any given day, 21.4% of the population consumed a beef sandwich. Among all Americans, beef sandwiches provided 6.3% of mean energy intake and accounted for approximately 10% of the population’s mean intake of vitamin B_12_ and saturated fat, 9% of protein and sodium, 7% of iron, 6% of choline, and 5% of potassium. Among beef sandwich consumers, beef sandwiches accounted for 26.2% of mean energy intake on a day of consumption. The beef component of sandwiches accounted for the majority of vitamin B_12_, choline, and protein, non-beef components accounted for the majority of sodium, iron, and potassium, and beef and non-beef components made similar contributions to saturated fat. Hamburgers provided consumers the lowest energy, sodium, and saturated fat intake, while non-burger sandwiches provided the highest intake of these nutrients.

**Discussion:**

Beef sandwiches are an important source of energy, protein, vitamin B_12,_ iron, and choline, and like other sandwiches, are also a source of sodium and saturated fat. Americans could enhance nutrient contributions from sandwiches by selecting lean meat and limiting use of saturated fat- or sodium-rich non-beef components.

## 1 Introduction

Sandwiches are commonly consumed foods in the United States (U.S.), with approximately 50% of all adults reporting consumption of a sandwich on any given day (1, 2). In an assessment of nutrient contributions from 36 subcategories of foods, “sandwiches” are the leading source of energy for the U.S. population at 14.8% of total intake, and a top source of several nutrients and food groups (3).

A dictionary definition of “sandwich” is a food item with “two or more slices of bread or a split roll having a filling in between” (4). In practice, the term sandwich may be used to more broadly refer to a variety of handheld meals with an outer bread or starch product (e.g., sliced bread, a split roll, a tortilla, or a taco shell) and a filling of one or more components typically including meat, poultry or fish, cheese, vegetables, condiments, and a spread or sauce. In dietary pattern analyses in the United States, sandwiches commonly are defined to represent the traditionally defined sandwiches (i.e., bread or a roll and a filling) as well as burritos and tacos (3).

The U.S. population consumes a wide variety of sandwiches, including but not limited to burger sandwiches, chicken/turkey sandwiches, egg/breakfast sandwiches, frankfurter sandwiches, cheese sandwiches, peanut butter and/or jelly sandwiches, burritos, and tacos. In this analysis, beef burger sandwiches are referenced simply as burgers (i.e., hamburgers and cheeseburgers). Previous analyses show that among adults, cold cut sandwiches (e.g., luncheon meat, deli meat, other sliced sandwich meat), burgers, and poultry sandwiches are the most commonly consumed sandwich (2). Burgers represent a primary form in which fresh beef [i.e., unprocessed or uncured beef as defined for estimating U.S. nutrient intakes (5)] is consumed in the U.S., while processed beef is consumed in some cold cuts or other cured meats, frankfurters, and sausages (6, 7). Beef therefore is an important component of many commonly consumed sandwiches. Beef also is recognized as a primary source of protein and several micronutrients in the U.S. diet (8, 9).

Despite the widespread consumption of sandwiches in the U.S. diet, we are not aware of an assessment of nutrient contributions from burgers or other beef-containing sandwiches. The purpose of this study was to examine the intake of energy and select nutrients from beef-containing sandwiches categorized as burgers (with subcategories of hamburgers and cheeseburgers) and non-burger sandwiches, and to estimate percentage contributions to total nutrient intakes from sandwiches and the beef and non-beef components.

## 2 Methods

### 2.1 Data source and study population

The data source for this analysis was the 2013–2014 and 2015–2016 What We Eat in America (WWEIA) dietary recall component of the National Health and Nutrition Examination Survey (NHANES), a nationally representative, cross-sectional survey (10). The first 24-h dietary recall was conducted by trained dietary interviewers using the Automated Multiple Pass Method, which is grounded in research-based strategies to enhance dietary recall of all foods consumed in a 24-h period, including foods consumed at home and away from home (11). The study population for this analysis was limited to individuals ages ≥2 years (excluding breastfeeding children) who provided day one dietary recalls determined to be reliable by the National Center for Health Statistics.

### 2.2 Identifying beef-containing sandwiches

In this analysis, beef sandwiches were identified as food codes for beef-containing sandwiches reported in the dietary recalls, or as reported intakes of sandwich combinations that contained beef. Consistent with the approach used by USDA in dietary intake analyses (3), burritos and tacos were categorized as sandwiches. Tortilla combinations were reviewed and categorized as a beef sandwich if the combination included a beef-containing burrito or taco with additions, or beef with a tortilla or other wrap. Burgers with or without cheese were categorized as cheeseburgers and hamburgers, respectively. All other beef sandwiches, which included beef hot dogs, beef corn dogs, beef burritos, beef tacos, beef steak sandwiches, corned beef sandwiches, pastrami sandwiches, beef deli meat sandwiches, meatball submarine sandwiches, and sloppy Joes, were categorized as non-burger beef sandwiches. Sausages and hot dogs consumed as a sandwich and identified as unspecified meat or mixtures with beef (e.g., “frankfurter or hot dog sandwich, meat and poultry, plain, on white bun”) were not classified as beef, and consequently were not captured in the assessment.

### 2.3 Identifying nutrients in beef and non-beef components in beef sandwiches

The levels of energy and select nutrients per 100 g of each beef sandwich and the beef component in beef sandwiches were quantified using survey-specific Food and Nutrient Data for Dietary Surveys (FNDDS) resources developed by USDA for processing nutrient intakes and supporting nutrient composition data (12, 13). In this analysis, the non-beef component refers collectively to the starch (e.g., bread, bun, tortilla, taco shell) and any fillings other than beef such as cheese, vegetables, condiments, sauces, and spreads. When FNDDS data for beef sandwiches provided line item data for beef and non-beef components, the data were categorized accordingly (beef vs. total non-beef components) and summed by food code. When the FNDDS data did not include individual line items for beef and non-beef components in a beef sandwich, the FNDDS data were critically reviewed and values for beef and non-beef components were systemically derived for relevant beef sandwich food codes. The amount of beef per 100 g sandwich was estimated as the percentage weight of the Food Patterns Equivalents Data (FPED) meat component in the sandwich relative to the FPED meat component in a 100% beef ingredient defined by the Food Patterns Ingredient Data (FPID) (14). FPID ingredient codes used for imputations of the beef components in this analysis corresponded to identified ingredient codes used by USDA in processing nutrient data for similar sandwiches, or represented ingredient codes that allowed for close approximation of nutrient totals for the sandwich. The nutrient profile for each beef ingredient was derived by multiplying the weight of the ingredient in the food code and the nutrient data for the ingredient. For example, in calculating the percentage weight of beef in codes for hamburgers that did not include a line item for beef, the weight of the beef component in hamburgers was calculated by dividing the FPED meat component in the sandwich by the FPED mean component in the FPID code (code number 13317) for “Beef, ground, patties, frozen, cooked, broiled.” Different FPID codes were selected to represent different types of beef in sandwiches. USDA nutrient retention factors were used in the calculations of nutrients from beef to account for changes in the nutrient composition of a food during cooking (15). This approach for calculating the weight percentage of beef and nutrients from beef accounted for the full nutrient profile of the beef ingredient, including both lean and fat constituents, and was not limited to lean meat equivalents. Sodium added to beef during preparation (per FNDDS data) was captured as a beef nutrient. The nutrient content of the non-beef components in all relevant sandwiches was calculated as the difference between the total nutrients provided by the sandwich and nutrients attributed to the beef component.

The current analysis was limited to nutritional components for which the 2020–2025 Dietary Guidelines for Americans (DGA) recognize sandwiches as a top source (energy, protein, saturated fat, and sodium) (16), and select micronutrients for which beef is recognized as a key source and the nutrient is underconsumed at one or more life stages (vitamin B_12,_ choline, iron, and potassium) (8, 16). Intakes of vitamin B_6_ and phosphorus, nutrients which are underconsumed by adolescents (16), were examined in children ages 2-18 years.

### 2.4 Analysis

Population characteristics of sex, age, race/ethnicity, and poverty income ratio were summarized and compared for beef sandwich consumers and non-consumers of beef sandwiches using the Pearson Chi-square test. Intake of energy and select nutrients from the total diet, beef sandwiches and the beef and non-beef components of beef sandwiches were calculated for each respondent by multiplying the food code-specific energy and nutrient content data by the gram weight of each reported food item and summing contributions for all components. Estimates of mean intake were developed on a *per capita* and per user basis by type of beef sandwich consumer (any beef sandwich, hamburger, cheeseburger, non-burger beef sandwich) where *per capita* intakes reflect intakes by consumers and non-consumers of beef sandwiches, and the per user estimates reflect intakes by individuals consuming any beef sandwich on day 1 of the NHANES dietary recall. Estimates were developed for the population ages 2 years and older and subpopulations of children ages 2–18 years (including separate analyses for males and females), younger adults ages 19–59 years, and older adults ages 60 years and older in accordance with the life-stage groups outlined in the 2020–2025 DGA. Statistical weights were used in all analyses so the results are representative of the U.S. population. Values are presented as means ± standard error (SE). Mean gram weight and nutrient intakes were calculated with Microsoft Access and error terms and statistical comparisons of demographic characteristics were developed with Stata version 12.1 (StataCorp LP, College Station, Texas). As this was a descriptive analysis, no statistical tests of dietary intakes were conducted.

## 3 Results

### 3.1 Characteristics of beef sandwich consumers

Consumption of beef sandwiches varied across all demographic characteristics of sex, age, race/ethnicity, and poverty income ratio (Table 1). On any given day, 21.4% of the population ages 2 years and older consumed a beef sandwich (Table 2). Non-burger beef sandwiches were consumed by the largest proportion of individuals (13.0%), followed by cheeseburgers (6.8%), and hamburgers (2.7%).

**Table 1:** Characteristics of beef sandwich consumers vs. non-consumers of beef sandwiches in the United States, 2013–2016.

Demographic group^a^	Beef sandwich consumers^b^	Non-consumers of beef sandwiches	*p*-value
Sample population, *n*			–
Any beef sandwich	3,355	12,629	
Non-burger	2007	13,977	
Cheeseburger	1,079	14,905	
Hamburger	462	15,522	
Sex, %			<0.01
Male	57.6 (1.4)	46.6 (0.48)	
Female	42.4 (1.4)	53.4 (0.48)	
Age, %			0.02
2–18 y	24.8 (1.21)	22.3 (0.75)	
19–59 y	56.9 (1.42)	56.2 (0.98)	
60+ y	18.2 (0.86)	21.5 (0.92)	
Race/Ethnicity, %			<0.01
Mexican American	14.2 (2.12)	9.8 (1.5)	
Other Hispanic	5.9 (0.84)	6.5 (0.82)	
Non-Hispanic White	63.1 (3.28)	61.5 (2.64)	
Non-Hispanic Black	11.6 (1.77)	12 (1.42)	
Non-Hispanic Asian	2 (0.36)	6.3 (0.84)	
Other Race - Including Multi-Racial	3.1 (0.33)	4 (0.36)	
Ratio of family income to poverty, %			0.01
Under 1.31	23.2 (1.45)	24.3 (1.63)	
1.31–3.50	37.2 (1.29)	32.9 (1.24)	
Over 3.50	39.6 (2.04)	42.8 (1.98)	

a Values other than sample counts presented as means (standard error).

b Consumers of beef burger sandwiches (hamburgers, cheeseburgers) and non-burger beef sandwiches, where non-burgers include all non-burger beef sandwiches such as beef hot dogs, beef corn dogs, beef burritos, beef tacos, beef steak sandwiches, corned beef sandwiches, pastrami sandwiches, beef deli meat sandwiches, meatball submarine sandwiches, and sloppy Joes.

**Table 2:** *Per capita* and per user nutrient intakes from beef sandwiches among the total U.S. population ages 2 years and older, 2013–2016.

Nutrient^a^	Total beef sandwiches^b^			Hamburgers			Cheeseburgers			Non-Burger Sandwiches^c^		
	Total diet	Sandwich^f^	Beef only	Total diet	Sandwich^f^	Beef only	Total diet	Sandwich^f^	Beef only	Total diet	Sandwich^f^	Beef only
*Per Capita*^d^	*N* = 15,984											
Gram weight	–	55 (1.9)	18 (0.5)	–	5 (0.4)	2 (0.2)	–	15 (0.8)	6 (0.3)	–	36 (1.8)	10 (0.4)
Energy, kcal	2064 (12.5)	129 (4.2)	48 (1.4)	–	12 (0.9)	6 (0.4)	–	38 (2.1)	16 (0.9)	–	79 (3.7)	26 (1.1)
Protein, g	79.5 (0.65)	6.9 (0.22)	3.9 (0.12)	–	0.7 (0.05)	0.5 (0.04)	–	2.3 (0.13)	1.4 (0.08)	–	4.0 (0.18)	2.0 (0.08)
Saturated fat, g	26.7 (0.25)	2.7 (0.09)	1.3 (0.04)	–	0.2 (0.02)	0.2 (0.01)	–	0.9 (0.05)	0.5 (0.03)	–	1.6 (0.08)	0.7 (0.03)
Iron, mg	14.2 (0.11)	1.1 (0.03)	0.4 (0.01)	–	0.1 (0.01)	0.1 (0.00)	–	0.3 (0.02)	0.1 (0.01)	–	0.6 (0.03)	0.2 (0.01)
Choline, mg	318 (2.7)	18 (0.5)	12 (0.4)	–	2 (0.2)	2 (0.1)	–	6 (0.3)	4 (0.2)	–	10 (0.4)	6 (0.3)
Vitamin B12, mcg	4.88 (0.063)	0.50 (0.015)	0.41 (0.012)	–	0.06 (0.005)	0.06 (0.004)	–	0.20 (0.011)	0.16 (0.009)	–	0.23 (0.009)	0.18 (0.008)
Sodium, mg	3,410 (21.8)	297 (10.9)	69 (2.6)	–	20 (1.6)	5 (0.4)	–	78 (4.4)	18 (1.1)	–	199 (10.0)	47 (2.4)
Potassium, mg	2,520 (22.8)	124 (4.5)	56 (1.6)	–	11 (0.9)	6 (0.5)	–	33 (1.8)	18 (1.0)	–	81 (4.1)	32 (1.2)
Per user^e^	*N* = 3,355 (21.4%)			*N* = 462 (2.7%)			*N* = 1,079 (6.8%)			*N* = 2007 (13.0%)		
Gram weight	–	259 (4.3)	82 (1.7)	–	184 (7.0)	76 (3.1)	–	217 (4.4)	85 (2.0)	–	274 (5.9)	75 (2.0)
Energy, kcal	2,304 (20.5)	605 (9.1)	223 (4.4)	2,127 (76.0)	429 (16.5)	212 (9.6)	2,401 (48.0)	558 (10.7)	237 (5.3)	2,326 (23.1)	611 (11.1)	199 (5.1)
Protein, g	85.0 (1.12)	32.4 (0.55)	18.4 (0.38)	77.8 (2.72)	25.7 (0.92)	18.8 (0.78)	90.1 (1.62)	33.4 (0.70)	20.5 (0.50)	84.9 (1.36)	30.5 (0.68)	15.5 (0.43)
Saturated fat, g	32.7 (0.39)	12.6 (0.24)	6.2 (0.14)	26.9 (0.88)	7.2 (0.40)	6.0 (0.32)	34.9 (0.81)	12.5 (0.26)	6.7 (0.14)	33.4 (0.45)	12.7 (0.31)	5.4 (0.18)
Iron, mg	15.5 (0.23)	4.9 (0.08)	1.8 (0.04)	14.3 (0.48)	4.4 (0.19)	2.0 (0.11)	15.6 (0.43)	4.3 (0.12)	2.0 (0.06)	15.9 (0.32)	4.9 (0.11)	1.5 (0.04)
Choline, mg	323 (5.1)	85 (1.4)	58 (1.3)	307 (12.3)	71 (2.9)	57 (2.5)	344 (8.3)	89 (2.0)	66 (1.6)	318 (6.3)	78 (1.7)	49 (1.4)
Vitamin B12, mcg	5.92 (0.119)	2.32 (0.045)	1.90 (0.037)	5.59 (0.258)	2.33 (0.098)	2.19 (0.096)	7.04 (0.198)	2.96 (0.058)	2.38 (0.050)	5.53 (0.153)	1.78 (0.042)	1.42 (0.033)
Sodium, mg	3,768 (49.9)	1,391 (27.9)	323 (12.0)	3,089 (116.9)	733 (26.7)	172 (9.3)	3,784 (86.6)	1,150 (24.7)	260 (9.5)	3,959 (52.3)	1,530 (35.6)	359 (18.6)
Potassium, mg	2,574 (28.5)	583 (9.8)	264 (5.2)	2,475 (94.4)	399 (14.9)	232 (9.2)	2,579 (48.6)	481 (10.0)	266 (6.0)	2,616 (34.3)	622 (13.2)	246 (6.4)

a All values presented as means (standard error).

b Includes beef burger sandwiches (hamburgers, cheeseburgers) and non-burger beef sandwiches.

c Includes all non-burger beef sandwiches such as beef hot dogs, beef corn dogs, beef burritos, beef tacos, beef steak sandwiches, corned beef sandwiches, pastrami sandwiches, beef deli meat sandwiches, meatball submarine sandwiches, and sloppy Joes.

d *Per capita* values represent average intakes for both consumers of beef sandwiches and non-consumers of beef sandwiches (i.e., no reported beef sandwich intake on day 1 of the NHANES dietary recall).

e Per user values represent average intakes for consumers only of each beef sandwich category on day 1 of the NHANES dietary recall.

f Nutrient values for “sandwich” represent nutrient intakes from beef and non-beef components combined in the sandwich. The “beef only” data represent nutrients in the beef component.

### 3.2 *Per capita* energy and nutrient intake from beef sandwiches

**Figure 1:**
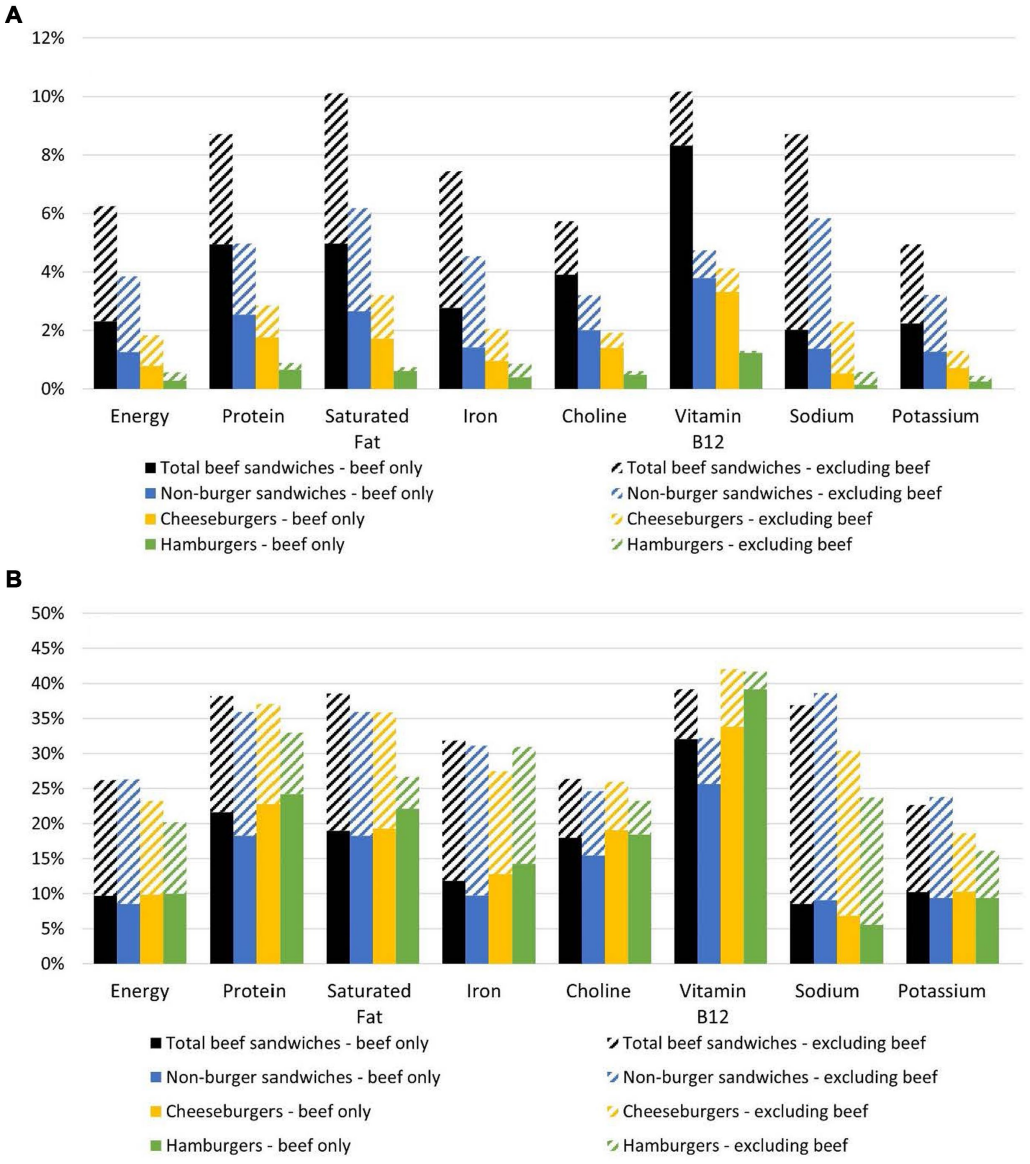
**(A)** *Per capita* percent contributions of beef sandwiches to nutrient intakes by the U.S. population ages 2 years and older (*n* = 15,984). Figure 1B. **(B)** Per user percent contributions of beef sandwiches to energy and nutrient intakes for the U.S. population ages 2 years and older: beef sandwich consumers (*n* = 3,355), hamburger consumers (*n* = 462), cheeseburger consumers (*n* = 1,079), and non-burger beef sandwich consumers (*n* = 2007). See Table 2 for energy and nutrient intakes by population. The “beef only” data represent nutrients in the beef component of the specified beef sandwich type, and the “excluding beef” component represents nutrients in all non-beef components collectively of the specified beef sandwich type. Total beef sandwiches include hamburgers, cheeseburgers, and non-burger beef sandwiches. Non-burger beef sandwiches include sandwiches such as beef hot dogs, beef corn dogs, beef burritos, beef tacos, beef steak sandwiches, corned beef sandwiches, pastrami sandwiches, beef deli meat sandwiches, meatball submarine sandwiches, and sloppy Joes.

Beef sandwiches provided a mean energy intake of 129 kcal for the total population ages 2 years and older (Table 2), which corresponds to 6.3% of mean energy intake from the total diet, and 0.6, 1.8, and 3.9% of mean energy intake from hamburgers, cheeseburgers, and non-burger beef sandwiches, respectively (Figure 1A). Beef sandwiches accounted for approximately 10% of the total population’s mean intake of vitamin B_12_ and saturated fat, 9% of protein and sodium intake, 7% of iron intake, 6% of choline intake, and 5% of potassium intake (Figure 1A).

### 3.3 Per user energy and nutrient intake from beef sandwiches

Among consumers of beef sandwiches ages 2 years and older, the mean energy intake from beef sandwiches on a day of intake was 605 kcal (26.2% of 2,304 kcal) (Table 2, Figure 1B), and beef sandwiches accounted for 37–39% of protein, saturated fat, vitamin B_12_ and sodium intake, one-third of iron intake, and roughly one-quarter of choline and potassium intake. Total energy and nutrient intakes and percentage contributions to intake varied by type of beef sandwich consumed. Among the three sandwich types, hamburgers accounted for the lowest per user percentage of total energy intake. Relative to non-burgers, burgers accounted for a higher percentage of vitamin B_12_ intake and lower percentages of total sodium and potassium intake.

### 3.4 Energy and nutrient contributions from beef vs. non-beef components in beef sandwiches

On a *per capita* and per user basis, the beef component in hamburgers, cheeseburgers, and non-burger beef sandwiches accounted for approximately 50, 40, and 33% of the mean energy provided by each sandwich (Figures 1A,B). The beef component in beef sandwiches accounted for the majority of vitamin B_12_, choline, and protein provided by the sandwich and the non-beef component accounted for the majority of sodium, iron, and potassium. Saturated fat contribution was dependent on sandwich type (Figure 1; Table 2). On a *per capita* and per user basis, the non-beef components (e.g., condiments, cheese, bun) in non-burger beef sandwiches accounted for more than 50% of the saturated fat intake from these sandwiches. The non-beef components also accounted for slightly more than 50% of the saturated fat intake from all beef sandwiches. The beef component accounted for slightly more than 50% of saturated fat from cheeseburgers and the majority of saturated fat from hamburgers, although the absolute intake of saturated fat from hamburgers was approximately 60% of the saturated fat intake from other beef sandwiches.

### 3.5 Energy and nutrient contributions from beef vs. non-beef components in beef sandwiches for other population groups

Results for children, younger adults, and older adults are provided in the Supplementary material. Relative to patterns of beef sandwich consumption for the population ages 2 years and older, patterns of consumption for younger adults ages 19–59 years were generally similar; on a *per capita* basis, 21.6% of younger adults consumed a beef sandwich and beef sandwiches accounted for 6.6% of total energy intake by this subpopulation. Among children ages 2–18 years, 23.2% of the subpopulation consumed a beef sandwich on a given day and these sandwiches accounted for 5.9% of total energy intake. Among adults ages 60 years and older, 18.7% of the subpopulation consumed a beef sandwich on a given day and these sandwiches accounted for 5.4% of total energy intake. Beef sandwiches also were a source of phosphorus and vitamin B_6_ in the diets of children. With the exception of hamburgers, the non-beef component accounted for the majority of phosphorus from beef sandwiches consumed by children, while the beef component accounted for the majority of vitamin B_6_ from beef sandwiches.

## 4 Discussion

Sandwiches are well recognized as a staple meal in the U.S. diet, but limited information is available on patterns of consumption for specific varieties of sandwiches. The current study provides descriptive information on the intake of energy and select nutrients from beef sandwiches categorized as hamburgers, cheeseburgers, and non-burger beef sandwiches for the U.S. population, and thus serves to inform public understanding on consumption of one category of sandwiches. Findings from this descriptive study show that on a given day, approximately one in five Americans consumes a beef sandwich. Among beef-containing sandwiches, non-burger sandwiches are most commonly consumed. Overall, beef sandwiches are an important source of energy and protein, and select nutrients of concern including vitamin B_12,_ iron, and choline. Beef sandwiches are also a notable source of sodium and saturated fat.

Sandwiches are a popular food item typically consumed as a meal (2, 17, 18), so their prominent contributions to energy and select nutrient intakes is not surprising. Beef is recognized as a rich source of protein and select micronutrients in the U.S. diet (8, 19), and these results show that the beef component provided the majority of vitamin B_12_, protein, and choline from beef sandwiches, while the non-beef component accounted for the majority of sodium, potassium, and iron. Although beef is a concentrated source of heme iron, most breads in the U.S. are made from enriched wheat flour and thus are a key dietary source of non-heme iron (20). Iron contributions from beef, which are highly bioavailable as heme iron, nonetheless are important for the subpopulation of women of childbearing age for whom iron is a nutrient of concern (16, 21).

Previous assessments have identified the sandwich category as a substantial contributor to sodium and saturated fat intake in the U.S. diet (2, 16). The 2020–2025 DGA reported that sandwiches accounted for 21% of total sodium intake and 19% of total saturated fat intake for the U.S. population ages 1 year and older, and the top ranking source of these two nutrients of concern (16). Findings from the current assessment, which like the 2020–2025 DGA was also based on NHANES 2013–2016 data, suggest that beef sandwiches accounted for approximately one-half of sodium and saturated fat intake from all sandwiches. The non-beef components of beef sandwiches provided the majority of sodium intake while the beef and non-beef components from all beef sandwiches combined made similar contributions to saturated fat intake, though relative contributions did vary by sandwich type. Common components in sandwiches overall and specifically beef-containing sandwiches including bread, cheese, and condiments (e.g., ketchup, salsa) are among the leading dietary sources of sodium intake (22–25). Cheese, beef, and processed meats are also leading sources of saturated fat intake (26). Results from this assessment of the population ages 2 years and older show that among the three types of beef-containing sandwiches, hamburgers provided consumers the lowest energy, sodium, and saturated fat intake, while non-burgers provided the highest intake of these nutrients. The non-burger beef sandwiches category represents a wide variety of sandwiches, including burritos and tacos, beef deli meat sandwiches, and hot dogs on a bun. Consumption of specific types of non-burger beef sandwiches and their contributions to beef and nutrient intakes were not examined in this analysis, though results from an assessment using data collected in 2015–2018 suggest that the categories of beef “burritos and tacos” and “cold cuts and cured meats” are greater sources of beef ounce equivalents (5.8 and 5.6%, respectively) among adults relative to other non-burger sandwich types (e.g., “frankfurter sandwiches” at 4.1% of beef ounce equivalents, or “other sandwiches” at 1.9% of beef ounce equivalents) (6). Burritos and tacos are commonly consumed with components including but not limited to various meats (e.g., beef, chicken, pork) or fish, cheese, beans, rice, sour cream, guacamole, and other condiments such as salsa or hot sauce, all of which contribute to total energy, sodium, and saturated fat provided by these sandwiches. In building any sandwich, many Americans could strive for healthier options including leaner cuts of meat as recommended in dietary guidance (16), and limited use of higher saturated fat- or sodium-rich components such as cheese, condiments, or other processed meat (e.g., bacon). The addition of fresh vegetables and use of whole grain breads in place of refined grain breads could also improve the nutrient profile of many sandwiches.

It is important to acknowledge that the term “sandwich” does not have a universal definition. In this analysis of beef sandwich consumption, identification of sandwiches aligned with a definition previously recognized in the U.S. It must be acknowledged that the lack of a standardized definition for a sandwich may limit interpretation of these data outside the U.S. The data herein were developed to inform U.S. dietary guidance but can also spur international groups to complete similar analyses with their own country-specific data and sandwich terminology, which could be a future research recommendation, given the popularity of these handheld meals.

Major strengths of the current analysis include use of a large, nationally representative sample of the U.S. population and identification of beef sandwiches (hamburgers, cheeseburgers, and non-burgers) reported as both single food codes and as consumption of beef in sandwich and tortilla or taco combinations. Disaggregation of sandwiches into beef and non-beef components allowed for examination of entire nutrient contributions from beef and non-beef components. The disaggregation of beef sandwiches accounted for the full nutrient profile of the beef ingredients and was not limited only to lean meat equivalents, though some values were imputed when the survey data files did not identify the beef component as a line item. It is also important to consider other limits of this study. This study is a descriptive analysis reflecting 1 day of self-reported recalls. The study was limited to energy and nutrient intakes exclusively from beef sandwiches. Given the focus on beef sandwiches in this brief analysis and specifically burgers, which are often called out as a specific type of sandwich that implies consumption of beef, further categorization and analysis of other types of sandwiches such as burritos/tacos, deli sandwiches, and hot dogs/frankfurters was not completed but warrants review in a larger study. Consideration of the environmental impact of select foods or dietary patterns was not within the scope of this study.

Findings from this descriptive study show that on a given day, beef sandwiches are an important source of energy and protein for the U.S. population, and a source of nutrients of concern for some subpopulations, including iron, choline, and vitamin B_12_. Beef sandwiches, like other sandwiches, are also a source of sodium and saturated fat. The beef component in beef sandwiches provides the majority of protein, vitamin B_12_, and choline provided by these sandwiches, while the non-beef component accounts for the majority of sodium, iron, and potassium. Saturated fat is provided by both the beef and non-beef components in total beef sandwiches, cheeseburgers, and non-burger sandwiches. Among beef-containing sandwiches, non-burger sandwiches are most commonly consumed. Many Americans could further enhance the nutrient contributions from sandwiches by selecting lean cuts of meat and sparing use of saturated fat- or sodium-rich non-beef additions.

## Data availability statement

Publicly available datasets were analyzed in this study. This data can be found at: https://www.cdc.gov/nchs/nhanes/about_nhanes.htm; https://www.ars.usda.gov/northeast-area/beltsville-md-bhnrc/beltsville-human-nutrition-research-center/food-surveys-research-group/.

## Ethics statement

NHANES was conducted in accordance with the Declaration of Helsinki, and approved by NCHS Research Ethics Review Board (Continuation of Protocol #2011–17).

## Author contributions

MM: Data curation, Formal analysis, Funding acquisition, Methodology, Project administration, Visualization, Writing – original draft. MA: Data curation, Formal analysis, Visualization, Writing – review & editing. XB: Data curation, Formal analysis, Methodology, Writing – review & editing.
