# Supplementary material for: Consumption of beef sandwiches in the United States and contributions to intake of energy and select nutrients
Source: Front Nutr. 2024 Jun 10;11:1355490. doi: 10.3389/fnut.2024.1355490 (PMC11194417; doi:10.3389/fnut.2024.1355490)
Supplement: Supplementary file 1 [file Data_Sheet_1.pdf]

Supplementary Material

Consumption of beef sandwiches in the United States and contributions to intake of energy and select nutrients

Supplemental Table 1a. Per capita and per user nutrient intakes from beef sandwiches among children ages 2-18 years, 2013-2016

	Total beef sandwiches†			Hamburgers			Cheeseburgers			Non-Burger Sandwiches‡		
**Nutrient***	**Total Diet**	**Sandwich****	**Beef only**	**Total Diet**	**Sandwich****	**Beef only**	**Total Diet**	**Sandwich****	**Beef only**	**Total Diet**	**Sandwich****	**Beef only**
**Per Capita**[§]						N=5669						
Gram weight	-	45 (2.4)	16 (0.8)	-	5 (0.5)	2 (0.3)	-	13 (1.0)	5 (0.5)	-	28 (2.0)	9 (0.6)
Energy, kcal	1866 (17.0)	110 (5.9)	44 (2.4)	-	11 (1.3)	6 (0.7)	-	33 (2.7)	14 (1.3)	-	65 (4.5)	24 (1.6)
Protein, g	67.9 (0.81)	5.8 (0.33)	3.4 (0.19)	-	0.7 (0.09)	0.5 (0.07)	-	2.1 (0.17)	1.3 (0.12)	-	3.1 (0.24)	1.6 (0.12)
Saturated fat, g	25.1 (0.37)	2.3 (0.14)	1.2 (0.07)	-	0.2 (0.02)	0.1 (0.02)	-	0.7 (0.06)	0.4 (0.04)	-	1.4 (0.11)	0.7 (0.05)
Iron, mg	13.8 (0.21)	0.9 (0.05)	0.3 (0.02)	-	0.1 (0.01)	0.1 (0.01)	-	0.3 (0.02)	0.1 (0.01)	-	0.5 (0.04)	0.2 (0.01)
Choline, mg	251 (3.4)	15 (0.8)	11 (0.6)	-	2 (0.2)	2 (0.2)	-	5 (0.5)	4 (0.4)	-	8 (0.5)	5 (0.4)
Vitamin B12, mcg	4.63 (0.081)	0.42 (0.023)	0.35 (0.019)	-	0.06 (0.008)	0.06 (0.008)	-	0.18 (0.015)	0.14 (0.012)	-	0.18 (0.013)	0.15 (0.010)
Sodium, mg	2997 (28.1)	252 (13.9)	67 (4.5)	-	19 (2.3)	4 (0.6)	-	68 (5.4)	15 (1.2)	-	165 (11.3)	48 (3.7)
Potassium, mg	2121 (21.5)	101 (5.4)	50 (2.5)	-	10 (1.2)	6 (0.8)	-	29 (2.3)	16 (1.4)	-	63 (4.3)	28 (1.8)
Phosphorus, mg	1251 (14.6)	76 (4.3)	28 (1.5)	-	6 (0.8)	4 (0.5)	-	23 (1.9)	9 (0.8)	-	47 (3.6)	15 (1.0)
Vitamin B6, mg	1.73 (0.027)	0.09 (0.005)	0.05 (0.003)	-	0.01 (0.001)	0.01 (0.001)	-	0.03 (0.004)	0.03 (0.003)	-	0.04 (0.003)	0.02 (0.002)
**Per User**[‖]		N=1286 (23.2%)			N=173 (3.1%)			N=411 (7.4%)			N=781 (14.1%)	
Gram weight	-	195 (7.1)	69 (2.4)	-	147 (8.5)	66 (4.8)	-	173 (6.2)	70 (3.2)	-	198 (9.2)	62 (3.2)
Energy, kcal	1999 (35.8)	475 (17.1)	189 (7.0)	1875 (93.1)	357 (21.4)	179 (13.5)	2033 (69.0)	451 (15.8)	192 (8.8)	2027 (48.6)	466 (21.3)	170 (9.2)
Protein, g	72.4 (1.73)	25.1 (1.01)	14.6 (0.61)	70.8 (5.79)	22.4 (1.41)	16.5 (1.21)	75.9 (2.65)	27.7 (1.04)	16.9 (0.80)	72.0 (2.35)	21.8 (1.33)	11.5 (0.77)
Saturated fat, g	28.8 (0.66)	9.7 (0.45)	5.3 (0.22)	25.7 (1.40)	5.5 (0.42)	4.8 (0.38)	29.6 (1.13)	9.8 (0.38)	5.3 (0.24)	29.4 (0.83)	9.6 (0.57)	4.8 (0.31)
Iron, mg	14.5 (0.38)	3.9 (0.14)	1.5 (0.05)	13.3 (0.75)	3.7 (0.23)	1.7 (0.13)	14.5 (0.67)	3.7 (0.13)	1.7 (0.08)	15.1 (0.60)	3.7 (0.18)	1.2 (0.07)
Choline, mg	260 (7.6)	65 (2.5)	46 (2.1)	258 (19.5)	61 (4.2)	50 (3.8)	279 (13.7)	73 (3.0)	54 (2.4)	253 (9.5)	56 (3.1)	36 (2.8)
Vitamin B12, mcg	5.48 (0.184)	1.82 (0.069)	1.52 (0.058)	5.19 (0.273)	1.98 (0.145)	1.87 (0.139)	5.91 (0.288)	2.38 (0.094)	1.92 (0.084)	5.47 (0.307)	1.32 (0.077)	1.08 (0.064)
Sodium, mg	3240 (63.8)	1089 (40.7)	289 (17.4)	2749 (157.5)	606 (36.8)	141 (13.7)	3144 (91.6)	919 (37.4)	202 (13.3)	3435 (91.4)	1177 (47.9)	339 (25.6)
Potassium, mg	2199 (48.4)	438 (16.1)	216 (7.7)	2067 (133.0)	327 (19.4)	205 (14.6)	2208 (84.2)	387 (15.2)	216 (9.6)	2252 (66.9)	446 (22.4)	197 (11.3)
Phosphorus, mg	1307 (27.3)	330 (13.0)	121 (4.7)	1140 (72.6)	194 (11.4)	125 (9.2)	1294 (45.6)	317 (11.0)	121 (6.1)	1364 (38.6)	333 (18.2)	108 (6.5)
Vitamin B6, mg	1.76 (0.061)	0.37 (0.017)	0.23 (0.013)	1.60 (0.141)	0.29 (0.019)	0.23 (0.018)	1.88 (0.080)	0.47 (0.038)	0.35 (0.027)	1.77 (0.081)	0.29 (0.020)	0.15 (0.014)

*All values presented as means (standard error).

†Includes beef burger sandwiches (hamburgers, cheeseburgers) and non-burger beef sandwiches.

‡ Includes all non-burger beef sandwiches such as beef hot dogs, beef corn dogs, beef burritos, beef tacos, beef steak sandwiches, corned beef sandwiches, pastrami sandwiches, beef deli meat sandwiches, meatball submarine sandwiches, and sloppy Joes.

§ Per capita values represent average intakes for both consumers of beef sandwiches and non-consumers of beef sandwiches (i.e., no reported beef sandwich intake on day 1 of the NHANES dietary recall).

‖ Per user values represent average intakes for consumers only of each beef sandwich category on day 1 of the NHANES dietary recall.

**Nutrient values for “sandwich” represent nutrient intakes from beef and non-beef components combined in the sandwich. The “beef only” data represent nutrients in the beef component.

Supplemental Table 1b. Per capita and per user nutrient intakes from beef sandwiches among male children ages 2-18 years, 2013-2016

	Total beef sandwiches†			Hamburgers			Cheeseburgers			Non-Burger Sandwiches‡		
**Nutrient***	**Total Diet**	**Sandwich****	**Beef only**	**Total Diet**	**Sandwich****	**Beef only**	**Total Diet**	**Sandwich****	**Beef only**	**Total Diet**	**Sandwich****	**Beef only**
**Per Capita§**						N=2856						
Gram weight	-	57 (3.8)	20 (1.5)	-	6 (1.0)	3 (0.5)	-	16 (1.7)	6 (0.8)	-	35 (2.9)	11 (0.9)
Energy, kcal	2044 (22.6)	138 (9.7)	55 (4.2)	-	14 (2.5)	7 (1.3)	-	42 (4.8)	18 (2.2)	-	83 (7.0)	30 (2.6)
Protein, g	76.0 (1.28)	7.3 (0.57)	4.2 (0.34)	-	0.9 (0.16)	0.6 (0.12)	-	2.6 (0.30)	1.6 (0.19)	-	3.8 (0.37)	2.0 (0.18)
Saturated fat, g	27.5 (0.49)	2.9 (0.23)	1.6 (0.12)	-	0.2 (0.04)	0.2 (0.04)	-	0.9 (0.11)	0.5 (0.06)	-	1.7 (0.17)	0.9 (0.08)
Iron, mg	15.1 (0.26)	1.1 (0.08)	0.4 (0.03)	-	0.1 (0.03)	0.1 (0.01)	-	0.3 (0.04)	0.2 (0.02)	-	0.7 (0.06)	0.2 (0.02)
Choline, mg	279 (5.0)	19 (1.5)	13 (1.1)	-	2 (0.4)	2 (0.4)	-	7 (0.8)	5 (0.6)	-	10 (0.9)	6 (0.6)
Vitamin B12, mcg	5.26 (0.126)	0.53 (0.043)	0.44 (0.035)	-	0.08 (0.015)	0.07 (0.014)	-	0.22 (0.026)	0.18 (0.021)	-	0.23 (0.022)	0.19 (0.016)
Sodium, mg	3300 (45.5)	319 (22.4)	87 (7.6)	-	23 (4.2)	5 (1.0)	-	85 (9.8)	19 (2.3)	-	211 (17.8)	63 (6.4)
Potassium, mg	2321 (29.3)	128 (8.7)	63 (4.4)	-	13 (2.2)	8 (1.5)	-	36 (4.0)	20 (2.4)	-	79 (6.2)	35 (2.5)
Phosphorus, mg	1382 (19.7)	96 (7.4)	35 (2.6)	-	8 (1.4)	5 (0.9)	-	29 (3.4)	11 (1.4)	-	59 (5.6)	19 (1.5)
Vitamin B6, mg	1.94 (0.040)	0.11 (0.008)	0.07 (0.006)	-	0.01 (0.002)	0.01 (0.002)	-	0.04 (0.007)	0.03 (0.005)	-	0.05 (0.004)	0.03 (0.002)
**Per User‖**		N=693 (26.3%)			N=96 (3.5%)			N=229 (8.8%)			N=413 (15.8%)	
Gram weight	-	216 (9.1)	76 (2.9)	-	164 (11.3)	74 (7.1)	-	181 (9.1)	73 (4.4)	-	222 (12.0)	69 (3.6)
Energy, kcal	2164 (53.8)	526 (22.0)	210 (9.2)	2075 (122.1)	398 (28.9)	202 (19.0)	2186 (114.4)	473 (22.4)	201 (12.4)	2195 (69.5)	523 (28.2)	191 (11.2)
Protein, g	79.5 (3.01)	27.8 (1.29)	16.1 (0.73)	79.7 (8.95)	25.0 (2.05)	18.5 (1.79)	81.6 (4.24)	29.2 (1.49)	17.8 (1.12)	79.7 (3.63)	24.3 (1.64)	12.7 (0.74)
Saturated fat, g	31.2 (0.87)	10.9 (0.61)	5.9 (0.31)	28.1 (2.07)	6.3 (0.56)	5.5 (0.53)	32.5 (1.68)	10.4 (0.51)	5.6 (0.33)	31.5 (1.22)	10.9 (0.82)	5.5 (0.40)
Iron, mg	15.5 (0.42)	4.3 (0.18)	1.6 (0.07)	14.4 (0.71)	4.2 (0.31)	1.9 (0.18)	15.5 (1.02)	3.8 (0.20)	1.8 (0.11)	16.2 (0.63)	4.1 (0.22)	1.3 (0.07)
Choline, mg	286 (12.1)	72 (2.9)	50 (2.6)	290 (26.5)	68 (6.1)	56 (5.7)	304 (21.7)	77 (4.2)	57 (3.4)	280 (15.3)	62 (3.4)	40 (2.9)
Vitamin B12, mcg	6.01 (0.306)	2.03 (0.093)	1.69 (0.078)	5.49 (0.333)	2.24 (0.207)	2.12 (0.199)	6.37 (0.420)	2.51 (0.130)	2.02 (0.116)	6.10 (0.488)	1.48 (0.097)	1.20 (0.073)
Sodium, mg	3512 (104.1)	1213 (52.2)	330 (22.0)	3067 (229.9)	666 (52.5)	154 (21.4)	3362 (157.0)	961 (49.1)	210 (14.9)	3745 (137.7)	1332 (68.2)	396 (33.7)
Potassium, mg	2368 (75.7)	485 (20.6)	241 (9.4)	2233 (192.8)	365 (26.7)	229 (22.1)	2401 (134.0)	405 (22.2)	226 (13.4)	2429 (99.5)	500 (28.0)	223 (12.3)
Phosphorus, mg	1430 (44.6)	364 (16.7)	133 (5.4)	1258 (105.8)	215 (16.2)	139 (13.8)	1405 (80.3)	334 (16.0)	127 (8.3)	1505 (57.6)	371 (24.4)	120 (6.5)
Vitamin B6, mg	1.89 (0.085)	0.40 (0.022)	0.25 (0.017)	1.77 (0.211)	0.32 (0.029)	0.26 (0.028)	1.97 (0.119)	0.49 (0.055)	0.36 (0.040)	1.92 (0.107)	0.32 (0.018)	0.17 (0.011)

*All values presented as means (standard error).

†Includes beef burger sandwiches (hamburgers, cheeseburgers) and non-burger beef sandwiches.

‡ Includes all non-burger beef sandwiches such as beef hot dogs, beef corn dogs, beef burritos, beef tacos, beef steak sandwiches, corned beef sandwiches, pastrami sandwiches, beef deli meat sandwiches, meatball submarine sandwiches, and sloppy Joes.

§ Per capita values represent average intakes for both consumers of beef sandwiches and non-consumers of beef sandwiches (i.e., no reported beef sandwich intake on day 1 of the NHANES dietary recall).

‖ Per user values represent average intakes for consumers only of each beef sandwich category on day 1 of the NHANES dietary recall.

**Nutrient values for “sandwich” represent nutrient intakes from beef and non-beef components combined in the sandwich. The “beef only” data represent nutrients in the beef component.

Supplemental Table 1c. Per capita and per user nutrient intakes from beef sandwiches among female children ages 2-18 years, 2013-2016

	Total beef sandwiches†			Hamburgers			Cheeseburgers			Non-Burger Sandwiches‡		
**Nutrient***	**Total Diet**	**Sandwich****	**Beef only**	**Total Diet**	**Sandwich****	**Beef only**	**Total Diet**	**Sandwich****	**Beef only**	**Total Diet**	**Sandwich****	**Beef only**
**Per Capita§**						N=2813						
Gram weight	-	33 (2.1)	12 (0.7)	-	3 (0.4)	2 (0.2)	-	10 (0.9)	4 (0.5)	-	20 (2.1)	6 (0.7)
Energy, kcal	1683 (16.6)	81 (4.8)	32 (1.8)	-	8 (1.1)	4 (0.6)	-	25 (2.6)	11 (1.3)	-	48 (4.9)	17 (2.0)
Protein, g	59.7 (0.68)	4.3 (0.26)	2.5 (0.16)	-	0.5 (0.07)	0.4 (0.05)	-	1.5 (0.16)	0.9 (0.11)	-	2.3 (0.27)	1.2 (0.18)
Saturated fat, g	22.6 (0.38)	1.6 (0.10)	0.9 (0.05)	-	0.1 (0.02)	0.1 (0.02)	-	0.5 (0.06)	0.3 (0.04)	-	1.0 (0.10)	0.5 (0.06)
Iron, mg	12.5 (0.23)	0.7 (0.04)	0.3 (0.02)	-	0.1 (0.01)	0.0 (0.01)	-	0.2 (0.02)	0.1 (0.01)	-	0.4 (0.04)	0.1 (0.02)
Choline, mg	221 (3.4)	11 (0.7)	8 (0.5)	-	1 (0.2)	1 (0.2)	-	4 (0.4)	3 (0.3)	-	6 (0.7)	4 (0.6)
Vitamin B12, mcg	3.98 (0.071)	0.31 (0.018)	0.26 (0.015)	-	0.04 (0.006)	0.04 (0.006)	-	0.13 (0.014)	0.10 (0.012)	-	0.13 (0.016)	0.11 (0.014)
Sodium, mg	2684 (31.3)	184 (11.9)	47 (4.1)	-	14 (1.9)	3 (0.5)	-	51 (4.8)	11 (1.4)	-	119 (11.3)	32 (3.9)
Potassium, mg	1914 (23.4)	75 (4.9)	36 (2.3)	-	7 (1.0)	5 (0.7)	-	21 (2.2)	12 (1.4)	-	46 (5.0)	20 (2.5)
Phosphorus, mg	1117 (14.0)	56 (3.7)	21 (1.5)	-	4 (0.6)	3 (0.4)	-	17 (1.8)	7 (0.8)	-	35 (3.8)	11 (1.5)
Vitamin B6, mg	1.52 (0.026)	0.06 (0.006)	0.04 (0.004)	-	0.01 (0.001)	0.01 (0.001)	-	0.03 (0.004)	0.02 (0.003)	-	0.03 (0.005)	0.02 (0.003)
**Per User‖**		N=593 (20.0%)			N=77 (2.7%)			N=182 (5.9%)			N=368 (12.2%)	
Gram weight	-	167 (6.6)	58 (2.5)	-	124 (9.1)	56 (4.2)	-	160 (7.2)	65 (4.5)	-	167 (9.1)	51 (4.1)
Energy, kcal	1775 (46.3)	405 (15.3)	160 (6.6)	1608 (160.8)	303 (21.2)	148 (12.1)	1800 (58.6)	417 (20.0)	177 (13.2)	1803 (59.3)	390 (21.0)	142 (10.2)
Protein, g	62.8 (1.44)	21.4 (0.92)	12.6 (0.66)	58.8 (5.15)	18.9 (1.31)	13.7 (1.09)	67.0 (3.01)	25.4 (1.26)	15.7 (1.10)	61.8 (2.21)	18.5 (1.47)	9.9 (1.13)
Saturated fat, g	25.6 (0.93)	8.2 (0.33)	4.4 (0.19)	22.5 (3.46)	4.5 (0.40)	3.9 (0.35)	25.2 (1.24)	9.0 (0.48)	4.9 (0.38)	26.5 (1.14)	8.0 (0.44)	4.0 (0.28)
Iron, mg	13.2 (0.62)	3.4 (0.13)	1.3 (0.06)	11.8 (1.08)	3.1 (0.20)	1.4 (0.10)	13.0 (0.84)	3.4 (0.16)	1.6 (0.11)	13.7 (0.94)	3.1 (0.18)	1.0 (0.09)
Choline, mg	224 (6.0)	56 (2.6)	39 (2.2)	216 (26.9)	51 (3.7)	41 (3.4)	240 (13.2)	67 (3.6)	49 (3.3)	217 (6.6)	47 (3.9)	30 (3.6)
Vitamin B12, mcg	4.75 (0.132)	1.54 (0.067)	1.29 (0.059)	4.80 (0.394)	1.63 (0.128)	1.54 (0.121)	5.19 (0.368)	2.17 (0.115)	1.76 (0.109)	4.62 (0.248)	1.10 (0.088)	0.91 (0.081)
Sodium, mg	2871 (74.0)	921 (37.2)	234 (18.7)	2326 (213.1)	525 (37.0)	124 (14.2)	2811 (104.8)	855 (40.0)	189 (17.9)	3022 (97.8)	971 (44.0)	263 (24.4)
Potassium, mg	1969 (45.9)	373 (15.3)	183 (8.6)	1846 (145.4)	276 (18.7)	173 (13.1)	1913 (75.6)	359 (17.7)	200 (14.2)	2015 (60.2)	374 (23.4)	162 (14.1)
Phosphorus, mg	1140 (26.0)	283 (12.8)	104 (5.8)	982 (90.1)	166 (10.9)	105 (8.3)	1125 (37.2)	291 (13.7)	112 (8.3)	1175 (42.0)	283 (18.8)	92 (9.2)
Vitamin B6, mg	1.58 (0.056)	0.32 (0.022)	0.21 (0.016)	1.36 (0.122)	0.24 (0.017)	0.19 (0.015)	1.75 (0.112)	0.44 (0.041)	0.33 (0.030)	1.56 (0.086)	0.26 (0.029)	0.14 (0.023)

*All values presented as means (standard error).

†Includes beef burger sandwiches (hamburgers, cheeseburgers) and non-burger beef sandwiches.

‡ Includes all non-burger beef sandwiches such as beef hot dogs, beef corn dogs, beef burritos, beef tacos, beef steak sandwiches, corned beef sandwiches, pastrami sandwiches, beef deli meat sandwiches, meatball submarine sandwiches, and sloppy Joes.

§ Per capita values represent average intakes for both consumers of beef sandwiches and non-consumers of beef sandwiches (i.e., no reported beef sandwich intake on day 1 of the NHANES dietary recall).

‖ Per user values represent average intakes for consumers only of each beef sandwich category on day 1 of the NHANES dietary recall.

**Nutrient values for “sandwich” represent nutrient intakes from beef and non-beef components combined in the sandwich. The “beef only” data represent nutrients in the beef component.

Supplemental Table 2. Per capita and per user nutrient intakes from beef sandwiches among young adults ages 19-59 years, 2013-2016

	Total beef sandwiches†			Hamburgers			Cheeseburgers			Non-Burger Sandwiches‡		
**Nutrient***	**Total Diet**	**Sandwich****	**Beef only**	**Total Diet**	**Sandwich****	**Beef only**	**Total Diet**	**Sandwich****	**Beef only**	**Total Diet**	**Sandwich****	**Beef only**
**Per Capita[§]**						N=7064						
Gram weight	-	64 (2.9)	19 (0.8)	-	5 (0.5)	2 (0.2)	-	17 (1.3)	7 (0.5)	-	41 (2.5)	11 (0.6)
Energy, kcal	2216 (16.1)	147 (6.3)	53 (2.2)	-	11 (1.2)	5 (0.6)	-	45 (3.2)	19 (1.4)	-	91 (5.1)	28 (1.6)
Protein, g	86.8 (0.95)	7.9 (0.32)	4.4 (0.18)	-	0.6 (0.07)	0.5 (0.05)	-	2.7 (0.19)	1.6 (0.12)	-	4.6 (0.25)	2.3 (0.14)
Saturated fat, g	28.1 (0.30)	3.1 (0.13)	1.5 (0.07)	-	0.2 (0.02)	0.2 (0.02)	-	1.0 (0.08)	0.5 (0.04)	-	1.9 (0.11)	0.8 (0.05)
Iron, mg	14.5 (0.14)	1.2 (0.05)	0.4 (0.02)	-	0.1 (0.01)	0.1 (0.01)	-	0.3 (0.02)	0.2 (0.01)	-	0.7 (0.04)	0.2 (0.01)
Choline, mg	350 (3.8)	21 (0.9)	14 (0.6)	-	2 (0.2)	1 (0.2)	-	7 (0.5)	5 (0.4)	-	12 (0.7)	7 (0.4)
Vitamin B12, mcg	5.19 (0.097)	0.57 (0.022)	0.46 (0.019)	-	0.06 (0.006)	0.06 (0.006)	-	0.24 (0.017)	0.19 (0.014)	-	0.27 (0.015)	0.21 (0.012)
Sodium, mg	3696 (29.7)	340 (16.4)	73 (4.0)	-	18 (2.0)	4 (0.6)	-	94 (6.9)	21 (1.7)	-	228 (14.1)	47 (3.5)
Potassium, mg	2670 (26.1)	143 (6.9)	63 (2.6)	-	10 (1.1)	6 (0.6)	-	39 (2.9)	21 (1.5)	-	94 (6.0)	36 (2.1)
**Per User[‖]**		N=1512 (21.6%)			N=175 (2.3%)			N=542 (7.4%)			N=890 (13.1%)	
Gram weight	-	294 (5.5)	90 (2.1)	-	206 (10.2)	84 (4.3)	-	235 (5.5)	91 (2.5)	-	315 (8.0)	82 (2.8)
Energy, kcal	2536 (31.6)	682 (11.2)	244 (5.3)	2443 (127.6)	481 (23.9)	241 (14.4)	2630 (56.7)	605 (13.2)	255 (6.5)	2550 (40.2)	694 (15.3)	214 (6.7)
Protein, g	93.7 (1.49)	36.7 (0.62)	20.5 (0.49)	87.5 (3.47)	28.3 (1.25)	20.9 (1.09)	98.1 (2.05)	35.9 (0.87)	22.1 (0.63)	93.7 (1.87)	35.0 (0.84)	17.5 (0.66)
Saturated fat, g	35.4 (0.53)	14.4 (0.29)	6.8 (0.18)	29.5 (1.34)	8.5 (0.63)	7.0 (0.51)	38.1 (0.88)	13.7 (0.33)	7.3 (0.17)	36.0 (0.70)	14.5 (0.41)	5.8 (0.23)
Iron, mg	16.1 (0.34)	5.5 (0.10)	2.0 (0.05)	15.1 (0.65)	5.0 (0.32)	2.3 (0.18)	16.0 (0.63)	4.5 (0.14)	2.1 (0.08)	16.6 (0.45)	5.6 (0.13)	1.7 (0.06)
Choline, mg	360 (8.5)	97 (1.8)	65 (1.6)	350 (19.0)	79 (4.3)	62 (3.6)	378 (12.1)	97 (2.4)	71 (2.0)	355 (10.9)	90 (2.6)	56 (2.2)
Vitamin B12, mcg	6.41 (0.172)	2.63 (0.054)	2.13 (0.046)	6.36 (0.435)	2.62 (0.137)	2.46 (0.135)	7.68 (0.301)	3.21 (0.074)	2.58 (0.064)	5.86 (0.203)	2.04 (0.061)	1.61 (0.048)
Sodium, mg	4168 (75.8)	1575 (33.6)	336 (15.4)	3458 (189.2)	801 (35.4)	180 (14.6)	4179 (102.6)	1256 (29.9)	288 (13.4)	4363 (78.8)	1738 (48.3)	359 (22.5)
Potassium, mg	2749 (41.4)	664 (12.8)	291 (7.1)	2779 (143.2)	442 (19.8)	253 (12.1)	2735 (73.4)	522 (12.7)	288 (7.6)	2779 (47.8)	720 (18.2)	272 (9.7)

*All values presented as means (standard error).

†Includes beef burger sandwiches (hamburgers, cheeseburgers) and non-burger beef sandwiches.

‡ Includes all non-burger beef sandwiches such as beef hot dogs, beef corn dogs, beef burritos, beef tacos, beef steak sandwiches, corned beef sandwiches, pastrami sandwiches, beef deli meat sandwiches, meatball submarine sandwiches, and sloppy Joes.

§ Per capita values represent average intakes for both consumers of beef sandwiches and non-consumers of beef sandwiches (i.e., no reported beef sandwich intake on day 1 of the NHANES dietary recall).

‖ Per user values represent average intakes for consumers only of each beef sandwich category on day 1 of the NHANES dietary recall.

**Nutrient values for “sandwich” represent nutrient intakes from beef and non-beef components combined in the sandwich. The “beef only” data represent nutrients in the beef component.

Supplemental Table 3. Per capita and per user nutrient intakes from beef sandwiches among older adults ages 60 years and older, 2013-2016

	Total beef sandwiches†			Hamburgers			Cheeseburgers			Non-Burger Sandwiches‡		
**Nutrient***	**Total Diet**	**Sandwich****	**Beef only**	**Total Diet**	**Sandwich****	**Beef only**	**Total Diet**	**Sandwich****	**Beef only**	**Total Diet**	**Sandwich****	**Beef only**
**Per Capita[§]**						N=3251						
Gram weight	-	44 (3.9)	14 (0.9)	-	6 (1.0)	3 (0.4)	-	9 (1.4)	4 (0.6)	-	28 (3.2)	8 (0.8)
Energy, kcal	1868 (20.5)	101 (8.8)	39 (2.4)	-	14 (2.1)	7 (1.0)	-	24 (3.7)	10 (1.6)	-	63 (7.0)	22 (2.0)
Protein, g	72.4 (0.89)	5.5 (0.46)	3.2 (0.21)	-	0.9 (0.13)	0.6 (0.09)	-	1.4 (0.22)	0.9 (0.14)	-	3.2 (0.36)	1.7 (0.17)
Saturated fat, g	24.5 (0.37)	2.0 (0.21)	1.1 (0.07)	-	0.2 (0.03)	0.2 (0.03)	-	0.5 (0.09)	0.3 (0.05)	-	1.3 (0.16)	0.6 (0.06)
Iron, mg	13.6 (0.26)	0.9 (0.07)	0.3 (0.02)	-	0.1 (0.02)	0.1 (0.01)	-	0.2 (0.03)	0.1 (0.01)	-	0.5 (0.06)	0.2 (0.02)
Choline, mg	306 (4.2)	14 (1.0)	10 (0.7)	-	2 (0.4)	2 (0.3)	-	4 (0.6)	3 (0.4)	-	8 (0.9)	5 (0.6)
Vitamin B12, mcg	4.33 (0.093)	0.39 (0.027)	0.32 (0.021)	-	0.08 (0.011)	0.07 (0.011)	-	0.13 (0.019)	0.10 (0.016)	-	0.18 (0.018)	0.15 (0.015)
Sodium, mg	3088 (41.5)	230 (18.6)	62 (6.1)	-	26 (4.0)	7 (1.0)	-	48 (7.5)	11 (1.8)	-	156 (16.4)	45 (6.5)
Potassium, mg	2553 (32.8)	98 (8.6)	46 (3.1)	-	14 (2.2)	8 (1.2)	-	20 (3.0)	11 (1.8)	-	64 (7.4)	27 (2.6)
**Per User[‖]**		N=557 (18.7%)			N=114 (2.7%)			N=126 (6.8%)			N=336 (13.0%)	
Gram weight	-	236 (11.1)	77 (2.4)	-	181 (13.2)	71 (5.2)	-	214 (7.0)	82 (4.8)	-	248 (18.8)	72 (3.2)
Energy, kcal	1994 (42.9)	540 (24.0)	207 (7.4)	1819 (62.1)	406 (26.4)	194 (14.1)	2030 (63.0)	540 (22.1)	234 (13.0)	2034 (64.5)	551 (37.9)	189 (9.4)
Protein, g	74.8 (1.97)	29.2 (1.34)	17.0 (0.59)	67.6 (3.12)	24.3 (1.73)	17.5 (1.32)	79.5 (2.79)	32.3 (1.35)	20.2 (1.21)	74.9 (2.91)	28.0 (1.98)	14.7 (0.75)
Saturated fat, g	29.5 (0.78)	10.9 (0.69)	5.7 (0.26)	23.6 (1.04)	6.4 (0.46)	5.3 (0.40)	30.5 (1.11)	12.1 (0.65)	6.8 (0.40)	30.9 (1.19)	11.1 (1.06)	5.1 (0.34)
Iron, mg	14.8 (0.52)	4.6 (0.18)	1.7 (0.06)	13.9 (0.84)	4.1 (0.26)	1.8 (0.13)	15.8 (1.30)	4.2 (0.22)	1.9 (0.14)	14.7 (0.70)	4.6 (0.30)	1.4 (0.07)
Choline, mg	292 (7.8)	77 (2.7)	53 (2.0)	280 (15.0)	68 (5.0)	53 (4.0)	311 (12.6)	86 (4.1)	64 (3.9)	289 (11.5)	72 (4.0)	46 (2.8)
Vitamin B12, mcg	4.96 (0.211)	2.06 (0.085)	1.72 (0.065)	4.60 (0.340)	2.18 (0.160)	2.03 (0.155)	6.19 (0.493)	2.87 (0.134)	2.33 (0.130)	4.59 (0.244)	1.60 (0.078)	1.30 (0.059)
Sodium, mg	3236 (72.3)	1226 (40.6)	330 (30.3)	2771 (104.6)	738 (58.6)	187 (15.5)	3153 (107.0)	1089 (49.4)	243 (27.9)	3413 (113.7)	1360 (62.9)	389 (45.2)
Potassium, mg	2538 (49.6)	525 (23.8)	246 (8.7)	2338 (93.8)	392 (31.4)	221 (17.0)	2551 (91.5)	469 (18.2)	259 (15.4)	2601 (81.7)	559 (41.4)	234 (12.3)

*All values presented as means (standard error).

†Includes beef burger sandwiches (hamburgers, cheeseburgers) and non-burger beef sandwiches.

‡ Includes all non-burger beef sandwiches such as beef hot dogs, beef corn dogs, beef burritos, beef tacos, beef steak sandwiches, corned beef sandwiches, pastrami sandwiches, beef deli meat sandwiches, meatball submarine sandwiches, and sloppy Joes.

[§] Per capita values represent average intakes for both consumers of beef sandwiches and non-consumers of beef sandwiches (i.e., no reported beef sandwich intake on day 1 of the NHANES dietary recall).

[‖] Per user values represent average intakes for consumers only of each beef sandwich category on day 1 of the NHANES dietary recall.

**Nutrient values for "sandwich" represent nutrient intakes from beef and non-beef components combined in the sandwich. The "beef only" data represent nutrients in the beef component.

Supplemental Figure 1

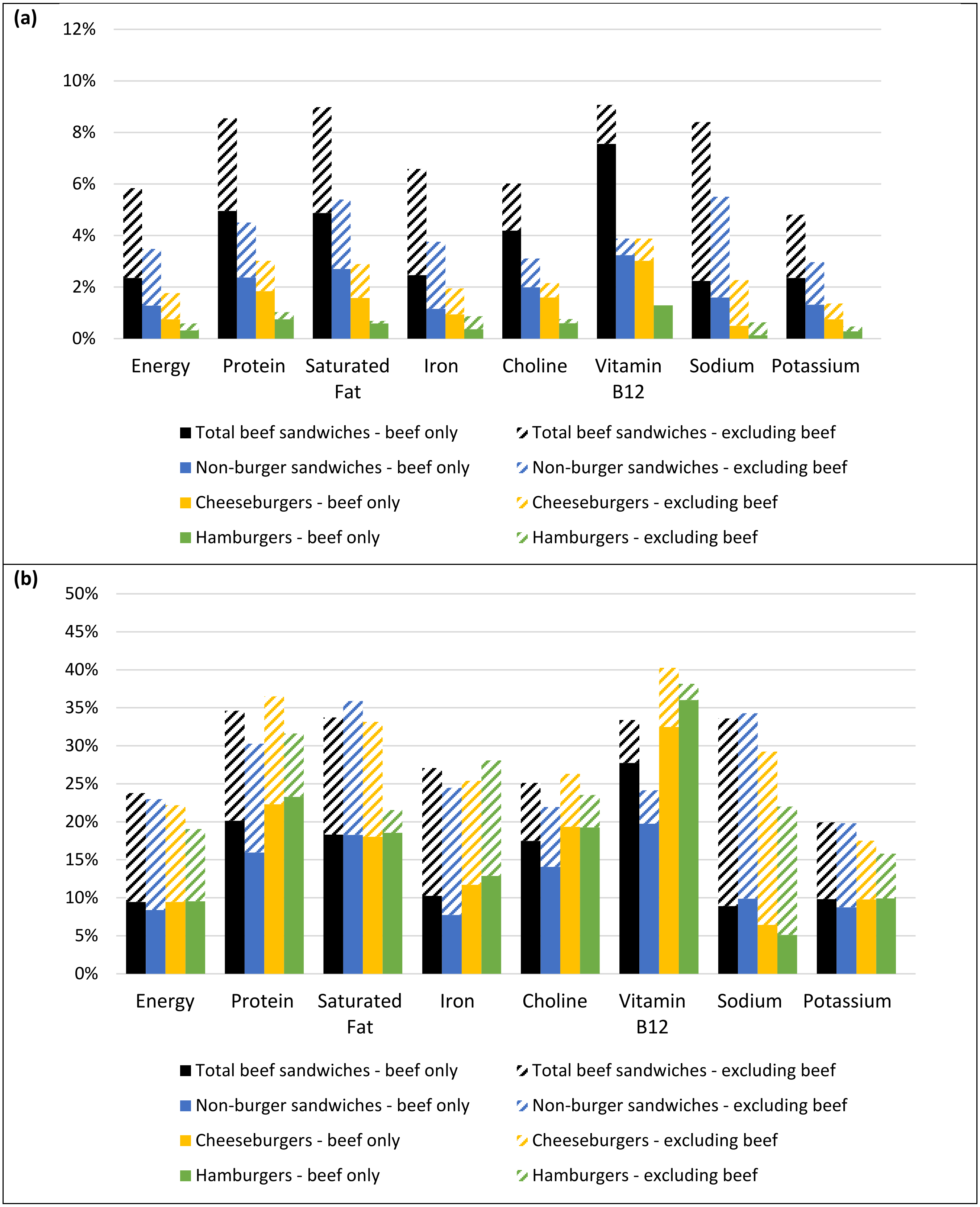

Supplemental Figure 1a. Per capita percent contributions of beef sandwiches to nutrient intakes by the U.S. population of children ages 2-18 years (n=5669). **Figure 1b.** Per user percent contributions of beef sandwiches to energy and nutrient intakes for the U.S. population of children ages 2-18 years: beef sandwich consumers (n=1286), hamburger consumers (n=173), cheeseburger consumers (n=411), and non-burger beef sandwich consumers (n=781). See Supplemental Table 1a for energy and nutrient intakes by the U.S. population of children ages 2-18 years. The "beef only" data represent nutrients in the beef component of the specified beef sandwich type, and the "excluding beef" component represents nutrients in all non-beef components collectively of the specified beef sandwich type. Total beef sandwiches include hamburgers, cheeseburgers, and non-burger beef sandwiches. Non-burger beef sandwiches include sandwiches such as beef hot dogs, beef corn dogs, beef burritos, beef tacos, beef steak sandwiches, corned beef sandwiches, pastrami sandwiches, beef deli meat sandwiches, meatball submarine sandwiches, and sloppy Joes.

Supplemental Figure 2

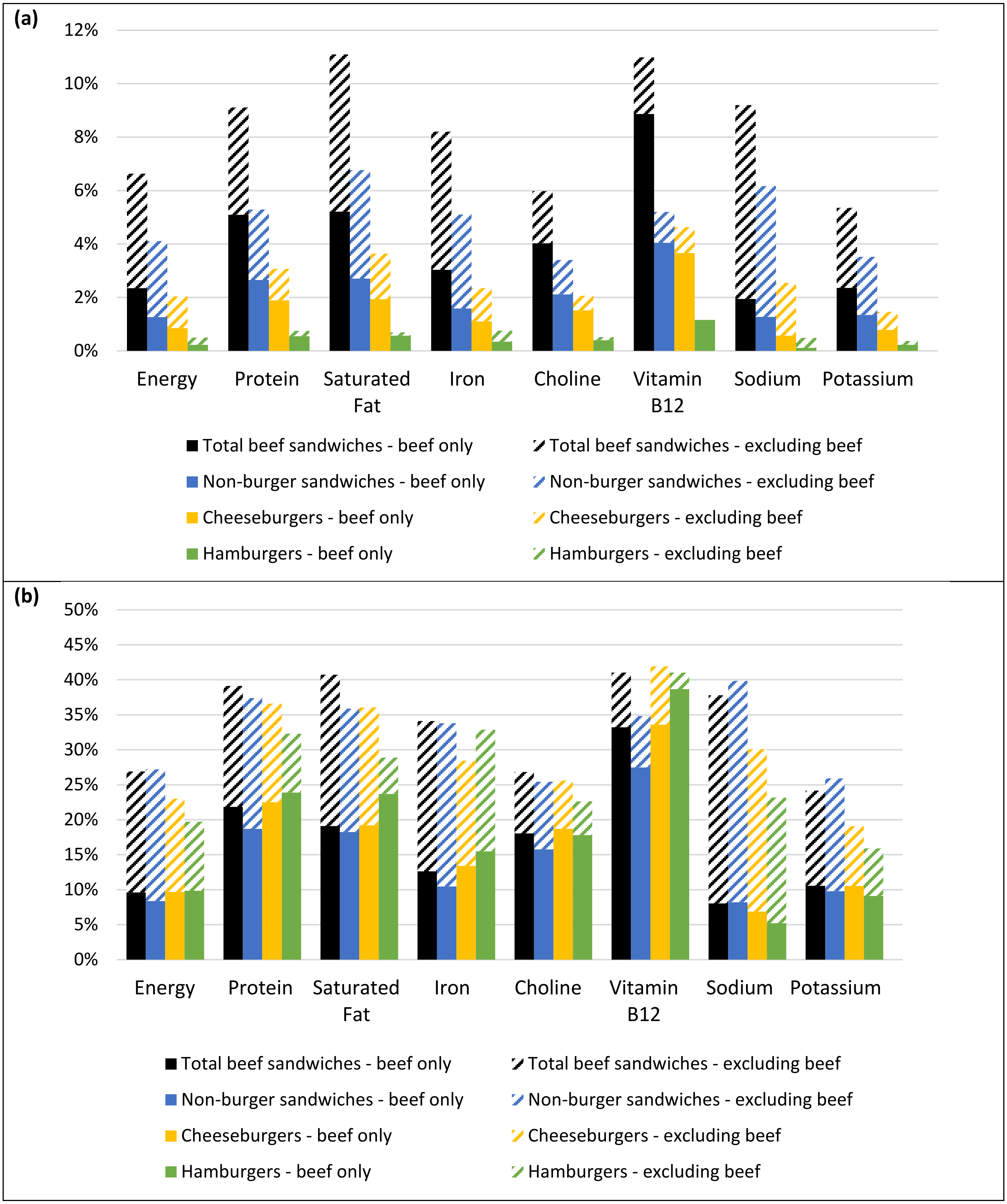

Supplemental Figure 2a. Per capita percent contributions of beef sandwiches to nutrient intakes by the U.S. population of young adults ages 19-59 years (n=7064). **Figure 2b.** Per user percent contributions of beef sandwiches to energy and nutrient intakes for the U.S. population of young adults ages 19-59 years: beef sandwich consumers (n=1512), hamburger consumers (n=175), cheeseburger consumers (n=542), and non-burger beef sandwich consumers (n=890). See Supplemental Table 2 for energy and nutrient intakes by the U.S. population of young adults ages 19-59 years. The "beef only" data represent nutrients in the beef component of the specified beef sandwich type, and the "excluding beef" component represents nutrients in all non-beef components collectively of the specified beef sandwich type. Total beef sandwiches include hamburgers, cheeseburgers, and non-burger beef sandwiches. Non-burger beef sandwiches include sandwiches such as beef hot dogs, beef corn dogs, beef burritos, beef tacos, beef steak sandwiches, corned beef sandwiches, pastrami sandwiches, beef deli meat sandwiches, meatball submarine sandwiches, and sloppy Joes.

Supplemental Figure 3

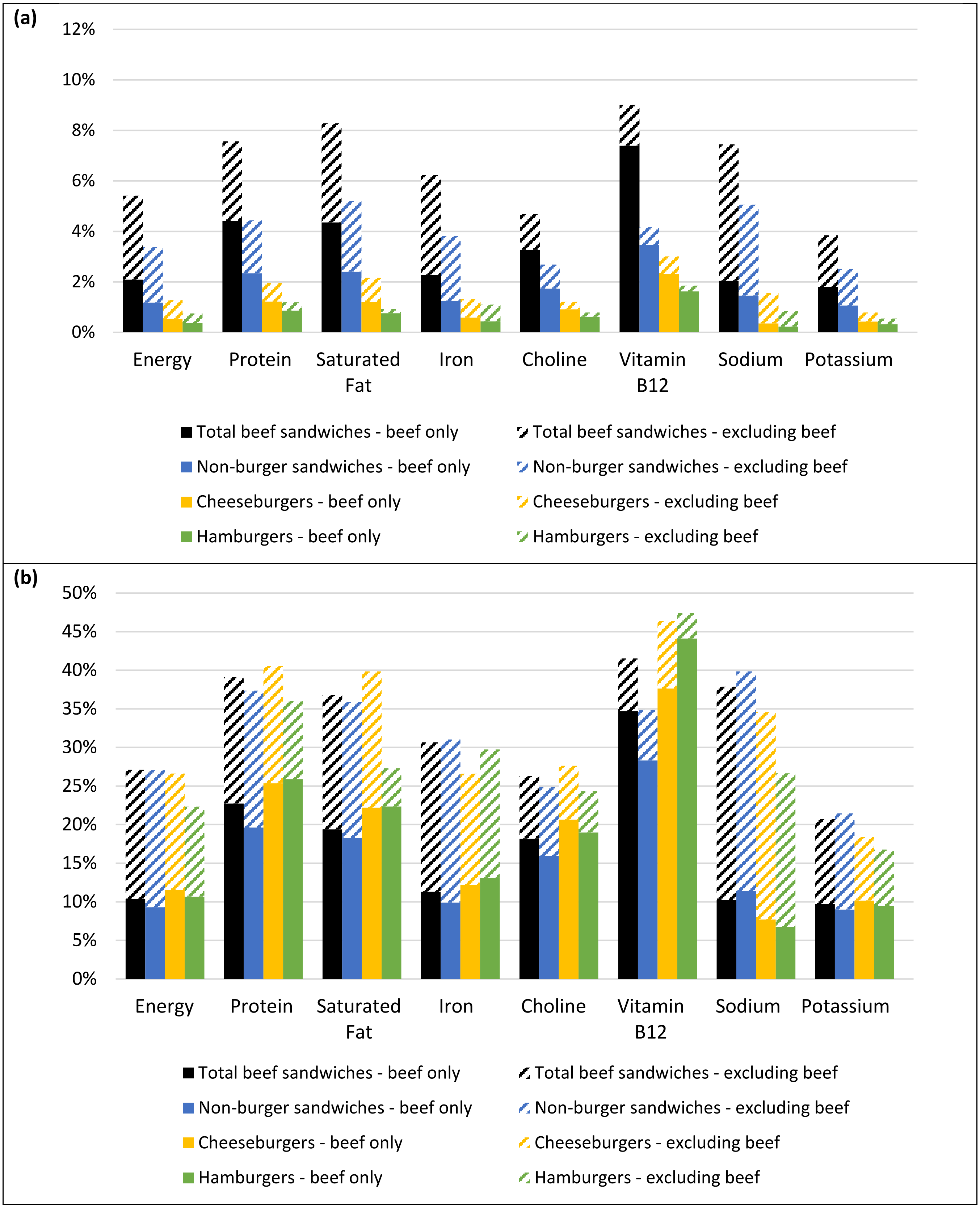

Supplemental Figure 3a. Per capita percent contributions of beef sandwiches to nutrient intakes by the U.S. population of older adults ages 60 years and older (n=3251). **Figure 3b.** Per user percent contributions of beef sandwiches to energy and nutrient intakes for the U.S. population of older adults ages 60 years and older: beef sandwich consumers (n=557), hamburger consumers (n=114), cheeseburger consumers (n=126), and non-burger beef sandwich consumers (n=336). See Supplemental Table 3 for energy and nutrient intakes by the U.S. population of older adults ages 60 years and older. The "beef only" data represent nutrients in the beef component of the specified beef sandwich type, and the "excluding beef" component represents nutrients in all non-beef components collectively of the specified beef sandwich type. Total beef sandwiches include hamburgers, cheeseburgers, and non-burger beef sandwiches. Non-burger beef sandwiches include sandwiches such as beef hot dogs, beef corn dogs, beef burritos, beef tacos, beef steak sandwiches, corned beef sandwiches, pastrami sandwiches, beef deli meat sandwiches, meatball submarine sandwiches, and sloppy Joes.
